# Nutrition, Safety, Health Functional Effects, and Availability of Honeybee (*Apis mellifera* L.) Drone Pupae

**DOI:** 10.3390/insects12090771

**Published:** 2021-08-27

**Authors:** Jae-Suk Choi

**Affiliations:** Department of Food Biotechnology, College of Medical and Life Sciences, Silla University, Busan 46958, Korea; jsc1008@silla.ac.kr; Tel.: +82-51-248-7789

**Keywords:** edible insects, entomophagy, novel foods, alternative foods, Hymenoptera

## Abstract

**Simple Summary:**

In July 2020, honeybee (*Apis mellifera* L.) drone pupae were registered as food ingredients in Korea. According to previous studies, this material has carbohydrates, fats, and proteins and contains amino acids, fatty acids, minerals, and vitamins, and is promising as an alternative food source. Prior studies have empirically demonstrated the microbiological and chemical safety of honeybee drone pupae. The health functional effects of this material have also been documented. This review describes the nutritional composition, safety, functionality, and availability of honeybee drone pupae reported so far. However, a comparison of the nutritional components of major livestock and other edible insects with those of honeybee pupae is needed. Future studies should continue to assess the allergic, parasitic, and other chemical hazards of honeybee drone pupae to ensure safety against unidentified hazards and investigate the functionality of honeybee drone pupae according to the criteria of traditional eastern medical texts. The review contributes to the literature because the citations it examined all empirically demonstrated and confirmed the nutritional value, food safety, and possible medicinal efficacy of honeybee drone pupa. This information could help increase profitability, improve apiary production sustainability, and generate alternative food and medicine sources.

**Abstract:**

Since ancient times, honeybee drone pupae have been used as food and for medicinal purposes in Asia, the United States, and Europe. Honeybee (*Apis mellifera* L.) drone pupae have been registered as food ingredients in Korea. This material is promising as an alternative food source. It has carbohydrates, fats, and proteins, and contains various amino acids and fatty acids as well as minerals and vitamins. Prior studies have empirically demonstrated the microbiological and chemical safety of honeybee drone pupae. The health functional effects of this material have been documented as well. However, to the best of my knowledge, no review has been conducted on the published studies to date. This review aimed to summarize the research findings on honeybee drone pupae thus far. Online databases were searched according to the selection criteria, duplicate reports were excluded, and 22 eligible articles were reviewed. Conclusionally, it was confirmed that honeybee drone pupae have various nutritional components, safety as a food and cosmetic material, and various available possibilities, but more systematic studies are needed to increase their consumption. Therefore, it is believed that this synopsis will help guide future research on honeybee drone pupae.

## 1. Introduction

Bees are globally distributed insects. Beekeeping is the oldest method of insect breeding [1]. In Korea, both Asian honeybees (*Apis cerana*) and Western honeybees (*Apis mellifera*) are raised in apiaries [2]. Honeybee colonies live in beehives constructed with numerous hexagonal chambers. A colony consists of worker bees, which are sterile females that constitute the majority of the colony, drone bees, which are male reproductive bees, and one queen bee capable of laying eggs. The queen bee creates new generations by breeding with the male drone bees. After mating, the drones are either expelled from the herd or killed to maintain the community. After the queen lays eggs and the larvae hatch, they are fed royal jelly provided by worker bees. A larger amount of royal jelly is provided for a longer period of time to the larvae that are expected to become queens [3].

The survey by Jeong et al. [4] indicated that there are 24,269 domestic beekeepers raising about 2.38 million bees, and annual sales of approximately 5.5 billion USD are made. However, honey production has decreased because of climate change. Hence, income has been destabilized in farm households, and the use of bee pupa as a food source is emerging [5].

Bee drones do not produce beekeeping products such as honey. Their exclusive purpose is mating with queen bees, and they consume the honey in the beehive. Farmers discard naturally occurring drone pupae as they have no commercial value [5]. Management of bee parasites of the genus *Varroa* is another common reason for drone brood removal from the beehive [6]. However, they can now serve as a novel income source for beekeepers [7,8]

Bee drones are sometimes used as nutritious, high-protein foods in Japan, China, the United States, and many European countries. They can be added to various dishes such as soups, confectioneries, and baked goods. They also serve as raw materials for chocolate, confectionery, alcoholic beverages, and functional foods [1,9,10]. Several studies have confirmed that the incorporation of ground insects into these familiar products (e.g., bread, biscuits, chocolate etc.) may be a good way to promote insects as food in markets in countries where entomophagy is not popular [11,12]. These findings can be used by the food industry to devise production and/or marketing strategies that overcome barriers to honeybee drone pupae consumption. The Food and Agriculture Organization has reported on the nutritional value, collection, storage, quality control, and use of bee larvae, pupae, and adult insects in food, medicine, and cosmetics [13]. Bee larvae (15.4%), pupae (18.2%), and adult bees (2.8%) have protein content comparable to those of beef (17.7%) and soybeans (12.9%) [13]. In July 2020, the Ministry of Food and Drug Safety and the Rural Development Administration of Korea recognized honeybee (*A*. *mellifera*) drone pupae as edible and a new food ingredient [14]. They contain carbohydrates, fats, and proteins [7]. Honeybee drone pupae that are 20–23 days old are harvested from the hive, and they are frozen immediately after harvesting and gathered for mass production [15]. Moreover, they are expected to be a new income source for beekeepers whose honey production has declined because of the reduced flowering period of black locust trees (*Robinia pseudoacacia* L.) due to climate change [16] and industrialization.

In recent years, numerous studies on the nutritional components, safety, functionality, and availability of bee drone pupae have been conducted in Korea. However, to the best of my knowledge, no systematic review has been performed on studies published on honeybee drone pupae to date. Therefore, this review aimed to summarize current research findings on honeybee drone pupae and propose future research directions for this material.

## 2. Materials and Methods

The literature search was conducted using online databases for papers written in English including Science Direct, PubMed, MEDLINE, and Web of Science. The DBpia, Earticle, and Korean studies Information Service System databases were consulted for papers written in Korean. The search strategy focused on the terms “*Apis mellifera*” and “drone OR pupae OR characteristics OR safety OR nutrition OR function OR effect.” Filters included in vitro, in vivo, and clinical trials. Bibliographies and references from the retrieved records were also considered. The relevant information was collected, duplicates were removed, and 22 articles were reviewed. Information on the nutritional composition, safety, functionality, and availability of honeybee (*Apis mellifera* L.) drone pupae was compiled and described.

## 3. Results

### 3.1. Nutritional Composition of Honeybee Drone Pupae

Six articles addressing the nutritional composition of honeybee drone pupae collected from various regions were identified. Proximate composition (Table 1) and amino acid, fatty acid, mineral, and vitamin contents were analyzed.

Kim et al. [5] evaluated the nutritional profile of 21–24 d honeybee (*Apis mellifera*) drone pupae collected in Changnyeong-gun and Gyeongnam-do, Korea, between April and June 2017. The moisture, crude protein, crude fat, carbonate, and ash concentrations in the proximate composition were 74.23 g/100 g, 11.05 g/100 g, 8.19 g/100 g, 5.68 g/100 g, and 0.85 g/100 g, respectively. Eighteen amino acids, including nine essential amino acids, were detected in the drone pupae. Glutamic acid had the highest concentration (1631.9 mg/100 g). Twelve minerals were identified in the drone pupae. Of these, K and P were the most abundant (235.78 mg/100 g and 177.35 mg/100 g, respectively). Vitamins B1, C, and E and fatty acids were present in low concentrations. The authors concluded that honeybee drone pupae are rich sources of proteins and other essential nutrients and could serve as a food ingredient.

Bee brood (pupae and larvae) was collected from the National Institute of Agricultural Sciences (NIAS; Wanju-gun, Jeollabuk-do, Korea) and analyzed by Choi et al. [7] for carbohydrate, saturated fatty acid, cholesterol, protein, fat, fiber, mineral, and vitamin contents. Bee brood was high in proteins (46.4–46.73 g/100 g), fats (18.84–20.75 g/100 g), carbohydrates (24.66–35.79 g/100 g), folic acid (222.30 µg/100 g), and vitamins. The folic acid content was markedly higher in the pupae than in the larvae. Bee brood was low in iron but a good source of folic acid and carbohydrates. The fats contained mainly saturated and monounsaturated fatty acids. The authors concluded that bee brood is an excellent source of several valuable nutrients, including lipids, carbohydrates, amino acids, essential minerals, and B vitamins. A high carbohydrate content is unusual in insects, and further research is necessary to understand this result in bee drones.

Kim et al. [17] analyzed the nutritional composition of 16th–20th instar drone pupae collected from the Jeonnam Agricultural Research & Extension Services (JARES; Naju-si, Jeollanam-do, Korea). In the freeze-dried bee pupa powder, the moisture, crude protein, crude fat, and crude ash levels were 1.69 ± 0.07 g/100 g, 48.52 ± 0.20 g/100 g, 23.41 ± 0.14 g/100 g, and 4.05 ± 0.02 g/100 g, respectively. The vitamin C and vitamin E concentrations were 14.92 ± 0.52 mg/100 g and 6.06 ± 0.11 mg α-TE/100 g, respectively. K and P were the most abundant minerals (1349.13 ± 34.57 mg/100 g and 1323.55 ± 43.85 mg/100 g, respectively). The Ca and Fe concentrations were 55.43 ± 1.51 mg/100 g and 5.49 ± 0.19 mg/100 g, respectively. The saturated and unsaturated fatty acid concentrations in the water-extracted, freeze-dried pupa powder were 59.62 g/100 g and 40.38 g/100 g, respectively. The palmitic (C16:0) and oleic (C18:1, n-9) fatty acid concentrations were 35.49 ± 0.08 g/100 g total fatty acids and 35.91 ± 0.22 g/100 g total fatty acids, respectively. The total amino acid content was 38.99 ± 2.63 g/100 g, and the free amino acid content was 5129.04 mg/100 g. The proline and glutamic acid concentrations were 1257.68 mg/100 g and 759.12 mg/100 g, respectively.

Bee drone pupae were collected every month between April and July 2020 from an apiary located in Wanju, Jeonbuk, Korea. The moisture, carbonate, crude protein, crude fat, and ash concentrations in the proximate composition of freeze-dried drone pupae were 0.23–0.76 g/100 g, 15.98–22.81 g/100 g, 51.87–53.92 g/100 g, 20.18–26.15 g/100 g, and 4.0–4.52 g/100 g, respectively [18].

The nutritional compositions of freeze-dried and hot air-dried honeybee drone pupae were compared. The pupae were between 17 and 23 d old and were collected from a beekeeping farm in Cheongyang-gun, Chungchungnam-do, Korea, in 2018 [19]. The moisture, crude protein, crude fat, crude ash, carbohydrate, and crude fiber concentrations in the freeze-dried honeybee drone pupae were 2.1 ± 0.02 g/100 g, 51.8 ± 0.15 g/100 g, 26.2 ± 0.13 g/100 g, 4.0 ± 0.06 g/100 g, 15.9 ± 0.15 g/100 g, and 2.7 ± 0.05 g/100 g, respectively. The moisture, crude protein, crude fat, crude ash, carbohydrate, and crude fiber concentrations in the hot air-dried honeybee drone pupae were 3.6 ± 0.03 g/100 g, 48.5 ± 0.21 g/100 g, 25.8 ± 0.11 g/100 g, 4.2 ± 0.04 g/100 g, 15.4 ± 0.17 g/100 g, and 2.5 ± 0.09 g/100 g, respectively. The protein content was 3.3 g/100 g higher in the freeze-dried powder than in the hot air-dried powder.

Ghosh et al. [20] assessed the nutritional composition of honeybee drone pupae from Korea and Denmark that were being evaluated as sustainable alternative food sources. Buckfast bee drones were collected from a healthy colony in an apiary located at the University of Copenhagen, Frederiksberg Campus, Copenhagen, Denmark, in the late summer of 2016. Italian bee drones were obtained from a healthy colony in an experimental apiary at Andong National University, South Korea, in the autumn of 2019. The fatty acid profiles of the Italian honeybee drone pupae from Korea (K-) and the Buckfast honeybee drone pupae from Denmark (D-) were measured. The saturated fatty acid (SFA; lauric acid, myristic acid, palmitic acid, stearic acid, arachidic acid, behenic acid, and lignoceric acid), monounsaturated fatty acid (MUFA; palmitoleic acid, elaidic acid, oleic acid, and *cis*-11-eicosenic acid), and polyunsaturated fatty acid (PUFA; linoleic acid, linolenic acid, and *cis*-13,16-docosadienoic acid) contents in the D-late pupae were 6634.61 mg/100 g, 5156.44 mg/100 g, and 67.87 mg/100 g (dry matter basis), respectively. The SFA, MUFA, and PUFA contents in the K-early pupae were 6414.11 mg/100 g, 4965.92 mg/100 g, and 99.20 mg/100 g, respectively. The SFA, MUFA, and PUFA contents in the K-late pupae were 5341.13 mg/100 g, 4470.72 mg/100 g, and 131.15 mg/100 g, respectively. The total fatty acid contents of the D-late pupae, K-early pupae, and K-late pupae were 11,858.92 mg/100 g, 11,479.23 mg/100 g, and 9,943.00 mg/100 g, respectively. The total amino acid contents of the D-late pupae, K-early pupae, and K-late pupae were 46.62 g/100 g, 42.56 g/100 g, and 49.39 g/100 g (dry matter basis), respectively. The mineral levels were also determined for the various pupae. The calcium concentrations in the D-late pupae, K-early pupae, and K-late pupae were 38.7 mg/100 g, 43.72 mg/100 g, and 49.29 mg/100 g (dry matter basis), respectively. The magnesium concentrations in the D-late pupae, K-early pupae, and K-late pupae were 81.86 mg/100 g, 82.89 mg/100 g, and 95.03 mg/100 g, respectively. The sodium concentrations in the D-late pupae, K-early pupae, and K-late pupae were 38.02 mg/100 g, 7.29 mg/100 g, and 8.52 mg/100 g, respectively. The potassium concentrations in the D-late pupae, K-early pupae, and K-late pupae were 1101.98 mg/100 g, 544.55 mg/100 g, and 643.06 mg/100 g, respectively. The phosphorus concentrations in the D-late pupae, K-early pupae, and K-late pupae were 802.61 mg/100 g, 774.03 mg/100 g, and 892.41 mg/100 g, respectively. The iron concentrations in the D-late pupae, K-early pupae, and K-late pupae were 5.99 mg/100 g, 4.86 mg/100 g, and 5.67 mg/100 g, respectively. The zinc concentrations in the D-late pupae, K-early pupae, and K-late pupae were 6.04 mg/100 g, 5.25 mg/100 g, and 5.88 mg/100 g, respectively. The copper concentrations in the D-late pupae, K-early pupae, and K-late pupae were 0.37 mg/100 g, 1.82 mg/100 g, and 1.94 mg/100 g, respectively. The manganese concentrations in the D-late pupae, K-early pupae, and K-late pupae were ND, 0.28 mg/100 g, and 0.29 mg/100 g, respectively. The total polyphenol and total flavonoid concentrations in the ethanol extracts of Buckfast honeybee drone pupae (Denmark) were 5.6 ± 0.2 mg/g, 4.7 ± 0.9 mg/g, and 4.5 ± 0.3 mg/g, respectively. The combined total sugar and reducing sugar concentration in the ethanol extracts of the drone pupae was 2.1 ± 0.2 mg/g.

### 3.2. Honeybee Drone Pupa Safety

The safety of honeybee drone pupa has been assessed based on heavy metal content [21], food pathogens and mycotoxins [18,19,22], residues of veterinary drugs [18], oxidative stability [23], and toxicity to dermal cells [24] (Table 2).

Choi et al. [21] analyzed the heavy metal content of honeybee drone pupae to establish safety guidelines for this new food material. Honeybee drone pupae were collected at apiaries in Yangpyeong, Gyeonggi-do, Cheongyang in 2018, Chungchungnam-do in 2018 and 2019, and Changnyeong, Gyeongnam-do in 2018, and then freeze-dried. As, Cd, Pb, and Hg concentrations were measured in the harvested material. No Cd was detected in the honeybee drone pupae from Yangpyeong, and only 0.001 mg/kg Cd was detected in the material collected from the other two regions. The Pb, Hg, and As concentrations were 0.02 mg/kg, 0.003 mg/kg, and 0.017 mg/kg, respectively, in the honeybee drone pupae from all three regions. According to the Korean Food Standards Codex, the levels of all measured heavy metals in the honeybee drone pupae were below the standard threshold values established for edible insects, namely, 0.1 mg/kg As, 0.05–0.3 mg/kg Cd, and 0.1–0.3 mg/kg Pb. Hence, regarding their heavy metal content, the sampled materials are safe to use as a food ingredient.

Another study measured the levels of harmful microorganisms and veterinary drugs such as neomycin, dihydrostreptomycin/streptomycin, bromopropylate, cymiazole, amitraz, oxytetracycline/chlortetracycline/tetracycline, coumaphos, flumethrin, and fluvalinate in honeybee drone pupae [18]. Drone pupae were obtained each month between April and July 2020 at an apiary located in Wanju, Jeonbuk, Korea. The Korean Food Code test methods were applied to detect coliforms, *Salmonella* spp., *Staphylococcus aureus*, and enterohemorrhagic *Escherichia coli*. None of these was detected during the honeybee drone pupa production season. Residues of six of the nine veterinary drugs were not detected in the material. Though cymiazole, amitraz, and fluvalinate were detected, their levels were below the maximum residue limits for these substances.

Kim et al. [22] analyzed harmful microorganisms and mycotoxins in honeybee drone pupae being considered for use as a food material. Honeybee drone pupae were collected from apiaries in Cheongyang, Chungchungnam-do in 2018, Gimje, Jeollabuk-do in 2018, and Changnyeong, Gyeongsangnam-do in 2017 and 2018, and immediately frozen and freeze-dried. The Korean Food Code test methods were applied to determine coliforms, *Salmonella* spp., *Staphylococcus aureus*, and enterohemorrhagic *E*. *coli*. None of these were detected in 280 honeybee drone pupae. Moreover, mycotoxins, aflatoxin B1, ochratoxin A, deoxynivalenol, and zearalenone were not detected in any sample. Therefore, honeybee drone pupae collected from beehives and promptly frozen are generally free of harmful microorganisms and mycotoxins and are safe to use as a food material.

Choi et al. [19] applied the Korean Food Standard Codex test methods to measure the abundance of coliforms, *Salmonella* spp., *Staphylcoccus aureus*, and enterohemorrhagic *E*. *coli.* None of these was detected either in freeze-dried or hot air-dried honeybee drone pupae powder [19].

Choi et al. [23] evaluated the oxidative stability of honeybee drone pupae to assess their fitness as a new edible food material that could be mass-produced in apiaries. Acid and peroxide values of 2.92 mg/g and 1.94 meq/kg, respectively, were obtained for 17–23 d honeybee drone pupae collected at Cheongyang (Chungchungnam-do), Gimje (Jeollabuk-do), and Changnyeong (Gyeongsangnam-do) in 2018. According to the Korean Food Codex, the processed edible pupa product standards are acid ≤ 5.0 mg/g and peroxide ≤ 60 meq/kg. Thus, these results indicate that honeybee drone pupae may be used as a new food material.

Honeybee drone pupae contain large amounts of high-grade fatty and amino acids. A toxicity evaluation of honeybee drone pupa extracts was conducted on skin cells to determine whether this material could be safely used in cosmetic formulations [24]. Honeybee drone pupae were collected from Jangseong-gun, Jeollanam-do, Korea, and extracted with 70% (*v*/*v*) EtOH. The extract was applied at various concentrations (≤25 μg/mL) to B16F10 (melanoma) and human dermal fibroblasts (HDF) cells. The MTT assay confirmed no cytotoxicity because the final cell viability was >90%. Three pupa extracts prepared using nine extraction methods were applied at various concentrations to human dermal papilla cells (HDPC), human keratinocytes (HaCaT), and mouse fibroblast cells (NIH3T3) and analyzed using MTS assay. No cytotoxicity was observed in HDPC treated with <50 μg/mL extract. When 5α-dihydrotestosterone (DHT) was co-applied to the cells with the pupae extracted with distilled water, 50% (*v*/*v*) EtOH, and 70% (*v*/*v*) EtOH, HDPC proliferation improved by 2–3%. HaCaT cells presented with no cytotoxicity under ≤2000 µg/mL extract. Cell proliferation was excellent for all treatment groups simultaneously subjected to pupa extract and lipopolysaccharide (LPS). No cytotoxicity was observed in NIH3T3 cells treated with <100 μg/mL extract. When these extracts were co-applied with tolbutamide to mitosis-inhibited cells, cell proliferation was promoted in the following order: distilled water extract >50% (*v*/*v*) EtOH extract >70% (*v*/*v*) EtOH extract.

### 3.3. Health Functional Effects of Honeybee Drone Pupae

Various health functional effects have been reported for honeybee drone pupae including antimicrobial [20,25], antioxidant [17,20,26], anti-inflammatory [27], antihyperglycemic [26], antidiabetic [28], antiobesity [29], platelet-aggregating [28], antiwrinkle [30], skin-whitening [30], hair loss prevention [31], and serum testosterone induction [32] activities (Table 3).

Lyophilized pupae aged 16–20 d were collected from Jangseong-gun, Jeollanam-do, Korea, and extracted with 5% (*v*/*v*) acetic acid. Antibacterial activity of the extracts against five types of dermatophytes was confirmed using the plate medium diffusion method [25]. The 5% (*v*/*v*) acetic acid honeybee drone pupa extract (100 mg/8 mm paper disk) had antimicrobial activity against *E. coli*, *Candida albicans*, and *Staphylococcus epidermidis* and created clear zones of 3.88 ± 1.55 mm, 0.86 ± 0.08 mm, and 7.08 ± 0.10 mm width, respectively. However, this extract had no inhibitory activity against the dandruff-causing yeast *Malassezia furfur* (also known as *Pityrosporum ovale*).

The antimicrobial activity of hot water and ethanol extracts of Buckfast honeybee drone pupae from Denmark was tested against pathogenic and food spoilage microorganisms including *Listeria monocytogenes*, *S. epidermidis*, *S*. *aureus*, *Bacillus subtilis*, *E. coli*, *Pseudomonas aeruginosa*, *Salmonella typhimurium*, *Proteus vulgaris*, *C. albicans*, and *Saccharomyces cerevisiae* [20]. The hot water and ethanol extract concentrations were 500 µg/disk and 1.0 µg/disk, respectively. None of the extracts displayed any antibacterial or antifungal activity. Kim et al. [17] evaluated the 1,1-diphenyl-2-picrylhydrazyl (DPPH) and the 2,2-azobis(3-ethylbenzothiazoline-6-sulfonate (ABTS+) radical scavenging activity of aqueous and ethanolic (50% (*v*/*v*), 70% (*v*/*v*), and 100% (*v*/*v*) EtOH) extracts of freeze-dried honeybee drone pupae collected from the JARES. The DPPH radical scavenging activity levels of 100 μg/mL DW, 50% (*v*/*v*) EtOH, 70% (*v*/*v*) EtOH, and 100% (*v*/*v*) EtOH extracts were 75.62%, 63.91%, 40.95%, and 9.38%, respectively. The ABTS+ radical scavenging activity levels of the same extracts were 57.09%, 84.48%, 82.48%, and 11.53%, respectively. Hence, honeybee drone pupa can be utilized as a new functional antioxidant material.

Kim et al. [26] evaluated the antioxidant activity of ethanolic extracts and various fractions of honeybee drone pupae collected at Cheongyang, Chungchungnam-do, in 2019. The ethyl acetate fraction (IC_50_ = 559.22 μg/mL) showed greater DPPH radical scavenging activity than the other samples (not detected). For the ABTS radical scavenging activity, the IC_50_ was in the range of 170.18–338.86 μg/mL. The butanol fraction (IC_50_ = 170.18 μg/mL) had approximately twice the ABTS radical scavenging activity of the hexane fraction (IC_50_ = 338.86 μg/mL).

The DPPH, ABTS, and nitrite scavenging activity and the reducing power of ethanol extracts of Buckfast honeybee drone pupae collected in Denmark in 2016 were also determined [20]. The concentration of the extract used for the DPPH, ABTS, and reducing power assays was 500 µg/mL, while that used for the nitrite scavenging assay was 200 µg/mL. The DPPH, ABTS, and nitrite scavenging activity levels of the late pupa ethanolic extracts were 1.3 ± 0.4%, 10.5 ± 0.5%, and 20.9 ± 4.1%, respectively. The reducing power (OD_700_) of the extracts was 0.008 ± 0.002. Adult drones exhibited the highest antioxidant activity, followed by the pupae and the larvae (data not shown), possibly because the polyphenol content increases with honeybee age.

Kim et al. [27] evaluated the inhibitory effect of honeybee drone pupa extract on nitric oxide (NO) production in LPS-challenged RAW264.7 macrophages. Honeybee drone pupae aged 17–23 d were collected at Cheongyang, Chungchungnam-do, in 2019, and methanolic extracts and their fractions were prepared using several solvents with different polarities. Neither the methanolic extracts nor their fractions were cytotoxic to the RAW264.7 cells. However, the hexane fraction lowered cell viability. All 50 μg/mL fractions, except butanol fraction, significantly inhibited NO production. The methanol, hexane, ethyl acetate, and butanol fractions reduced NO synthesis by 13.20%, 17.30%, 16.19%, and 1.61%, respectively.

Kim et al. [26] evaluated the dipeptidyl peptidase-4 (DPP-4) inhibitory activity of the ethanolic extracts and various fractions of honeybee drone pupae collected at Cheongyang, Chungchungnam-do, in 2019. The DPP-4 inhibitory effects of the ethanolic extract and the hexane, ethyl acetate, and butanol fractions had IC_50_ = 1242.50 μg/mL, 1491.65 μg/mL, 956.87 μg/mL, and 1196.57 μg/mL, respectively. The ethyl acetate fraction displayed the highest DPP-4 inhibitory activity.

Ethanolic extracts were prepared from freeze-dried honeybee drone larvae (DL), pupae (DP), and adults (DA) collected at Københans Universitet, Denmark, in 2016. In vitro antidiabetic activity was evaluated [28]. At 0.5 mg/mL, the honeybee DL, DP, and DA extracts showed 17.8%, 17.6%, and 17.4% inhibition of α-amylase level, respectively, and 5.8%, 7.2%, and 9.2% inhibition of α-glucosidase level, respectively. However, none of the honeybee extracts inhibited the β-amylase level.

The lipase activity levels were determined for hot air-dried and freeze-dried 17–23 d honeybee drone pupae [29]. A lipase activity assay kit was purchased from Abcam (Cambridge, UK) and used in this analysis. No lipase activity was detected in the control group. However, the lipase activity levels of the hot air-dried and freeze-dried pupae were 2.14 ± 0.27 mU/mL and 1.06 ± 0.06 mU/mL, respectively. Hence, the hot air-dried pupae had about twice the lipase activity of the freeze-dried pupae.

Ethanol extracts were prepared from honeybee DL, DP, and DA collected at Københans Universitet, Denmark, in 2016. In vitro platelet aggregation of these extracts was assessed [28]. At 0.25 mg/mL, the DL and DP extracts (but not the DA extract) showed strong collagen-induced platelet aggregation (235.7% and 240.7%, respectively). Therefore, DL and DP extracts could be used as styptic agents.

Kim et al. [30] assessed honeybee drone pupa (JARES) extracts for use in cosmetic materials. The anti-wrinkle activity of a 50% (*v*/*v*) EtOH extract of 16–20 d honeybee drone pupae (DPE) was evaluated by measuring collagen or collagenase gene expression in HDF. DPE at 100 μg/mL was not cytotoxic to HDF. Collagen type I expression increased by 46.7% and 66.7% in HDF subjected to 20 μg/mL and 100 μg/mL honeybee DPE, respectively. MMP1 collagenase expression decreased by 36.9% and 71.7% in HDF subjected to 20 μg/mL and 100 μg/mL honeybee DPE, respectively. Wrinkle reduction efficacy increased with DPE concentration in a dose-dependent manner. Thus, DPE is an effective antiwrinkle agent, as it increases collagen production and inhibits collagenase expression.

Kim et al. [30] demonstrated the skin-whitening effect of honeybee DPE (JARES) (50% [*v*/*v*] EtOH) via in vitro tyrosinase inhibition and B16F10 melanoma assays. For the skin-whitening test, the concentration of the positive control arbutin was set to 100 μg/mL. The concentration of the melanin-inducing m-melanocyte-stimulating hormone (α-MSH) was set to 100 nM. Matrix metalloproteinase-1 (MMP1) expression decreased in response to DPE in a concentration-dependent manner. The DPE treatment also inhibited melanin generation in B16F10 cells. For the in vitro tyrosinase inhibition test, 5 mg/mL honeybee DPE inhibited tyrosinase activity against *L*-tyrosine and L-DOPA by 40.7% and 53.4%, respectively. The B16F10 cells were treated with α-MSH to increase intracellular melanin production. Then, 100 μg/mL DPE was added, and the melanin content had significantly (*p* < 0.01) decreased to 41.7%. Overall, the skin-whitening effect increased with DPE concentration. Hence, DPE is an effective skin-whitening material, as it reduces melanin production.

Kim et al. [31] performed in vitro assays to evaluate the effects of 16–20 d honeybee DPE (50% (*v*/*v*) EtOH) on male pattern baldness, inflammatory hair loss, and hair growth. The honeybee drone pupae were acquired from the JARES. Human follicle dermal papilla cells, human keratinocytes (HaCaT), and mouse fibroblast cells (NIH3T3) maintained excellent proliferation in response to honeybee DPE treatment. Analysis of the inhibition of the genes regulating male pattern hair loss by DPE revealed that *cAMP* expression was downregulated by DHT and upregulated by DPE. *TGF-β1* inhibits cell division and differentiation, and its expression was strongly downregulated after ethanolic DPE exposure. However, this treatment also recovered *IGF-1* expression. The assay of the inhibitory effect of DPE on inflammatory hair loss showed that the expression of *TNF-α* and *IL-6* was significantly (*p* < 0.05 and *p* < 0.01, respectively) downregulated. The caspase-3 gene expression was significantly (*p* < 0.05) and most strongly repressed by 1000 μg/mL DPE. The assay of the inhibitory effect of DPE on the collagen type 1 gene confirmed that its expression was significantly (*p* < 0.01) induced when DPE was applied at 100 μg/mL. Overall, ethanolic DPE derived from honeybee drone pupae repressed the expression of genes related to hair loss and induced the expression of genes associated with hair growth. Therefore, DPE may help prevent hair loss and promote hair growth.

Weight change and testosterone levels were measured in male Sprague-Dawley rats subjected to 4 wks of oral administration of 250 mg/kg *Cordyceps militaris* produced from honeybee drone pupae. The aim was to evaluate the stimulatory efficacy of honeybee drone pupae containing large amounts of folic acid on serum testosterone levels [32]. Honeybee drone pupae were collected from the NIAS. The rat groups did not differ in body weight, food consumption amount, or water intake level. However, serum testosterone levels significantly (*p* < 0.05) increased in the rats treated with *C*. *militaris* grown on honeybee drone pupa medium (3.475 ± 0.750 pg/mL) or honeybee drone pupae (2.750 ± 0.843 pg/mL) relative to that of the control (2.225 ± 0.435 pg/mL). Therefore, fruiting bodies of *C*. *militaris* grown on the honeybee drone pupa medium and honeybee drone pupa powder may be efficacious in the treatment of reproductive disorders caused by low testosterone levels in human males.

### 3.4. Honeybee Drone Pupa Availability

Honeybee drone pupae are currently being used as a raw material for low-molecular-weight proteins [33], a sodium nitrite replacement [34], a medium for *Cordyceps* mushroom incubation [35], food and health functional food materials, and pharmaceutical and functional cosmetic agents.

Kim et al. [33] evaluated the availability of low-molecular-weight proteins generated from the hydrolysis of honeybee drone pupa proteins. This material could be used as a dietary or protein supplement for muscle development. The food proteinases alcalase (A), neutrase (N), and flavourzyme (F) were used to hydrolyze the proteins in honeybee drone pupae provided by the JARES. The order of the yield of low-molecular-weight proteins hydrolyzed by each enzyme was A+N > A+F > A > N > F > C. The overall yield of low-molecular-weight protein was improved by 128–165%, compared with that of the control. The hydrolysis level sharply increased for the first 2 h. F+N processing yielded the highest available amino acid concentration (4.15 mg/mL) after a 12–24 h hydrolysis. The protein patterns were confirmed using 14% sodium dodecyl sulfate-polyacrylamide gel electrophoresis (SDS-PAGE). The branched-chain amino acid content rapidly increased to a high level, especially in response to the F+N treatment. Therefore, the low-molecular-weight protein produced by the hydrolysis of honeybee drone pupa protein could be effective as a protein supplement consumed for dietary or muscle development purposes.

Kang et al. [34] examined the effects of substituting honeybee drone pupa meal (DPM) for sodium nitrite (SN) and vitamin C (VC) on the physicochemical quality characteristics of emulsion-type sausages. Honeybee drone pupae were collected at the NIAS. Samples were prepared with 150 ppm SN+200 ppm VC (control), 75 ppm SN+100 ppm VC+6.015% DPM (T1), or 12.03% DPM (T2), and stored at 4 ℃ for 30 d. The pH significantly (*p* < 0.05) decreased with increasing DPM concentration. The moisture and protein content significantly (*p* < 0.05) decreased, but the fat and ash content significantly (*p* < 0.05) increased with increasing DPM concentration. T1 and T2 had significantly (*p* < 0.05) higher saturated fatty acid content and significantly (*p* < 0.05) lower unsaturated and polyunsaturated fatty acid contents than those of the control. T1 and T2 had significantly (*p* < 0.05) lower L* and a* and significantly (*p* < 0.05) higher b* and h° than the control. C* was the lowest (*p* < 0.05) in T2. The TBARS content was the highest (*p* < 0.05) in T2 and twice that of the control (*p* < 0.05). T1 and T2 had significantly (*p* < 0.05) firmer textures than the control. These findings suggest that DPM does not effectively replace SN or VC and negatively affects color, lipid oxidation stability, and texture in emulsion-type sausage.

Hong et al. [35] developed a bee pollen agar (BPA) medium with drone powder and optimized its content to grow entomopathogenic chalkbrood fungi. Honeybee drone pupae were collected at the NIAS. Various honeybee drone pupa powder concentrations were used in the BPA medium. The growth rates of *Ascosphaera apis* var. *apis* (KACC 41774, 41775) and *A*. *apis* var. *major* (KACC 41610, 41778) mycelia were higher on BPA medium supplemented with 10% (*w*/*w*) honeybee drone pupa powder than they were on potato dextrose agar (PDA) medium. Moreover, the BPA medium was ideal for fungal isolation because the organism formed distinct radial growth lines in it. Entomopathogenic fungi have been used as biological control agents against insect pests. These fungi grew better on BPA medium supplemented with 5% (*w*/*w*) honeybee drone pupa powder than they did on PDA medium. Mycelia of Cordyceps mushrooms such as *Cordyceps scarabaeicola* have been widely used as traditional herbal medicines throughout East Asia. Their growth rates were higher on BPA medium supplemented with 2.5% (*w*/*w*) or 5.0% (*w*/*w*) honeybee drone pupa powder than they were on PDA medium. Hence, BPA medium supplemented with honeybee drone pupa powder could replace PDA medium for the propagation and isolation of entomopathogenic fungi.

## 4. Discussion

Recently, honeybee (*Apis mellifera* L.) drone pupae were registered as food ingredients in Korea. Since that time, basic research on this product has been actively progressing. Studies to date have shown that honeybee drone pupae are promising as edible insect products. However, to my knowledge, research regarding acceptance and life cycle assessment of honeybee drone pupae has not been performed. Further research is needed to overcome these problems.

The findings reported in the six articles addressing nutritional composition included in this review indicated that honeybee drone pupae are a rich source of proteins and other essential nutrients such as carbohydrates, fats, amino acids, minerals, and vitamins and could, therefore, be a valuable food ingredient. Future studies should determine whether the nutritional value of drone pupa varies with geographic region, habitat, harvest time, pupa age, and honey (nectar) source. Concerning geographic region, Ghosh et al. [20] compared the nutritional composition of honeybee drone pupae from Korea and Denmark. They confirmed that the nutritional composition of the honeybee pupa was different depending on the habitat. In addition, the nutritional composition of the honeybee pupa collected from various regions in Korea was reported [6,7,17,18]. Therefore, it is necessary to analyze the nutritional components of the honeybee drone pupa according to the geographic region through systematic studies in the future. Other substances in insects are beneficial for human health, for example, antibacterial proteins and peptides, enzymes, and hormones [36]. However, according to the review, there are no studies on these substances in the honeybee pupa. Therefore, it is necessary to conduct systematic research to identify these substances and their functionality.

The lower consumption of specific insect species could hamper the potential contribution of insects to food security [37]. In general, the allergic, microbial, parasitical, and chemical hazards of edible insect candidates should be evaluated to promote their consumption by humans [38,39]. The European Food Safety Authority (EFSA) Scientific Committee forms opinions based on risk profile and presents information on potential biological and chemical hazards as well as allergenicity and environmental hazards associated with farmed insects used as food and feed while taking into account the entire chain, from farming to the final product [40]. The findings reported in the six articles included in this review demonstrated the safety of honeybee drone pupae in terms of their microbial pathogen levels [18,19,22], heavy metal content [21], oxidation [23], and antibiotic residue levels [18]. Specifically, prior studies have validated the heavy metal, microbiological, and dermatological safety of honeybee drone pupa and demonstrated its potential as a food and cosmetic ingredient [18,19,21,22,23]. Future studies should continue to assess the allergic, parasitical, and other chemical hazards of honeybee drone pupae to ensure safety against unidentified hazards.

Various health functional effects of honeybee drone pupae have been reported, including antimicrobial [20,25], antioxidant [17,20,26], anti-inflammatory [27], antihyperglycemic [26], antidiabetic [28], anti-obesity [29], platelet-aggregating [28], anti-wrinkle [30], skin-whitening [30], hair loss prevention [31], and serum testosterone-inducing activities [32]. Functional verification of honeybee drone pupae has been performed mainly at the in vitro level using aqueous and ethanolic extracts and organic solvent fractions. Future studies should investigate the physiological activity of purified compounds isolated from honeybee drone pupae and applied clinically and in vivo.

According to the traditional Chinese medicine text Shinnongbonchogyeong (the first professional pharmacology text in China) [41], a honeybee drone pupa (*A*. *cerana*) is sweet and has a flat qi (spirit with energy). The medicinal effects of honeybee drone pupae have been described as follows: alleviation of headaches; removal of poison from snakes, centipedes, toads, flies, and so on; and compensation for damage to pale (wan) spleen, stomach, and liver deficient in blood [41]. It has been said that a person who takes honeybee drone pupa for a long time develops a shiny and healthy complexion and does not age.

The traditional Chinese medicine text Benchao Gangmu [42] states that honeybee (*A*. *cerana*) drone pupa has therapeutic efficacy against heart disease, jaundice, abdominal pain, vomiting, rubella, visceral bleeding, and hypoplasia. Moreover, honeybee drone pupae enhance immunity, prevent aging, promote recovery from physical weakness and fatigue, and treat intellectual development disorders in children [42].

According to the Korean traditional medicine text Donguibogam [43], long-term use of honeybee drone pupae (*A*. *cerana*) makes the complexion of a person shiny and healthy and delays aging. Hongjing Tao (Hong-gyeong Do in Korean; 456–536 C.E.) was a doctor and medical scientist during the North-South Dynasty of China. Hongjing Tao soaked honeybee drone pupa in liquor and applied it to the face to brighten and whiten the complexion. Honeybee drone pupae were used to treat symptoms such as chest and stomach pain, yellowish face and eyes, and vomiting in adults and children infected with five types of intestinal roundworms. Honeybee drone pupae were used to treat erysipelas, rubella, fever in the stomach, and difficulty urinating. They remove swelling, promotes lactation, and treat leucorrhea in women [43].

The honeybee drone pupa referred to by old Korean and Chinese documents is, in fact, the Asian *A*. *cerana* [44]. However, this review discussed and established the efficacy of the Western *A*. *mellifera*. *A*. *cerana* is a species of honeybee native to southern, southeastern, and eastern Asia. It was introduced into the Korean peninsula 2000 years ago. In 1910, *A*. *mellifera* was introduced into the Korean peninsula, and recently, most beekeepers in Korea are using this bee species [44]. Therefore, most of the recent research results obtained in Korea are those using the Western species of honeybee pupae. The pharmacological properties differ between the eastern and western honeybee drone pupa species. Hence, future research should validate the efficacy of the western honeybee drone pupa species in the same manner as it has already been determined for the eastern honeybee drone pupa species.

To the best of my knowledge, no study on the physiological activity of western honeybee drone pupa has been mentioned in any of the previous traditional Asian medical texts. Therefore, future studies should investigate the functionality of western honeybee drone pupa according to the criteria of traditional eastern medical texts, through in vitro, in vivo, and clinical studies. 

Edible insects have been mainly regarded as functional food materials and pharmaceutical and functional cosmetic agents. Although I included only three related articles in this review, the applicability of honeybee drone pupae besides these general uses has been investigated. Further studies are needed to develop various food additives and primary raw materials, including proteins, fats, and chitin from honeybee drone pupae.

## 5. Conclusions

Honeybee drone pupae are useful as a food resource and a promising novel functional food and pharmaceutical agent. In this review, I analyzed previous studies on honeybee drone pupae, thereby providing a direction for future studies to promote the human consumption of honeybee drone pupae.

## Figures and Tables

**Table 1:** Nutritional composition of honeybee (*Apis mellifera* L.) drone pupae.

Stage	Collecting Sites and Year	Results	Ref.
21–24 d drone pupae	in Changnyeong-gun and Gyeongnam-do, Korea, between April and June 2017	Moisture (74.23 g/100 g), crude protein (11.05 g/100 g), crude fat (8.19 g/100 g), carbonate (5.68 g/100 g), and ash (0.85 g/100 g)	[5]
Bee brood (pupae and larvae)	Wanju-gun, Jeollabuk-do in 2020	Protein (46.4–46.73 g/100 g), fat (18.84–20.75 g/100 g), and carbohydrate (24.66–35.79 g/100 g)	[7]
Freeze-dried 16th–20th instar drone pupae	Naju-si, Jeollanam-do, Korea	Moisture (1.69 ± 0.07 g/100 g), crude protein (48.52 ± 0.20 g/100 g), crude fat (23.41 ± 0.14 g/100 g) and crude ash (4.05 ± 0.02 g/100 g)	[17]
Freeze-dried drone pupae	Wanju-gun, Jeollabuk-do in 2020	Moisture (0.23–0.76 g/100 g), carbonate (15.98–22.81 g/100 g), crude protein (51.87–53.92 g/100 g), crude fat (20.18–26.15 g/100 g) and ash (4.0–4.52 g/100 g)	[18]
Between 17 and 23 d old pupae	Cheongyang-gun, Chungchungnam-do, Korea	Moisture (2.1 ± 0.02 g/100 g), crude protein (51.8 ± 0.15 g/100 g), crude fat (26.2 ± 0.13 g/100 g), crude ash (4.0 ± 0.06 g/100 g), carbohydrate (15.9 ± 0.15 g/100 g), and crude fiber (2.7 ± 0.05 g/100 g)	[19]

**Table 2:** Safety of honeybee (*Apis mellifera* L.) drone pupae.

Hazards	Collecting Sites and Year	Results	Ref.
Heavy metals: arsenic (As), cadmium (Cd), lead (Pb), and mercury (Hg)	Yangpyeong, Gyeonggi-do; Cheongyang in 2018, Chungchungnam-do in 2018 and 2019, and Changnyeong, Gyeongnam-do in 2018 in Korea	Cadmium was detected none or 0.001 mg/kg. Lead, mercury, and arsenic were detected at 0.02, 0.003, and 0.017 mg/kg in all three regions.	[21]
Harmful microorganisms	Wanju-gun, Jeollabuk-do in 2020	Coliforms, *Salmonella* species, *Staphylococcus aureus*, and enterohemorrhagic *Escherichia coli* were not detected	[18]
Veterinary drugs residues		When 9 veterinary drugs’ (neomycin, dihydrostreptomycin/streptomycin, bromopropylate, cymiazole, amitraz, oxytetracycline/chlortetracycline/tetracycline, coumaphos, flumethrin, and fluvalinate) residues were tested in drone pupae, cymiazole, amitraz and fluvalinate were detected, but the levels were mostly below the MRL (Maximum Residue Limit)	
Harmful microorganisms	Cheongyang, Chungchungnam-do in 2018, Gimje, Jeollabuk-do in 2018, and Changnyeong, Gyeongsangnam-do in 2017 and 2018 in Korea	Coliforms, *Salmonella* species, *Staphylococcus aureus*, and enterohemorrhagic *Escherichia coli* were not detected in 280 honeybee drone pupas	[22]
Mycotoxins		Mycotoxins, aflatoxin B1, ochratoxin A, deoxynivalenol, and zearalenone were not detected	
Harmful microorganisms	Cheongyang-gun, Chungchungnam-do in Korea	Coliforms, *Salmonella* spp. *Staphylcoccus aureus*, and enterohamorrhagice *Escherichia coli* were not detected in both freeze-dried and hot-air powder	[19]
Oxidative stability	Yangpyeong-gun, Gyeonggi-do; Cheongyang-gun, Chungchungnam-do and Changnyeong-gun, Gyeongnam-do in Korea in 2018	The acid value and the peroxide value of drone pupae were 2.92 ± 0.28 mg/g and 1.94 ± 0.26 m meq/kg, respectively	[23]
Toxicity evaluation on dermal cells	Jangseong-gun, Jeollanam-do, Korea. Not mentioned collecting year	No cytotoxicity observed by 25 μg/mL of 70% drone pupae EtOH extracts in B16F10 (Melanoma) and HDF (Human Dermal Fibroblasts) cells	[24]

**Table 3:** Health functional effects of honeybee (*Apis mellifera* L.) drone pupae.

Functionality	Extract or Fraction	Collecting Sites and Year	Activity	Ref.
Antimicrobial activities	Hot water and ethanol extracts	Buckfast honey bee drone (*A*. *mellifera*) pupae from Denmark	In 500 µg/disc and 1.0 µg/disc, no antibacterial and antifungal activities of hot water and ethanol extracts of Buckfast honey bee drone (*A*. *mellifera*) larvae, and pupae were noticed against pathogenic and food spoilage microorganisms.	[20]
Antimicrobial activities	5% acetic acid	Jangseong-gun, Jeollanam-do, Korea	In 100 mg/8 mm paper disc, antimicrobial activity against *Escherichia coli*, *Candida albicans*, and *Staphylococcus epidermidis* showing 3.88 ± 1.55 mm, 0.86 ± 0.08 mm, and 7.08 ± 0.10 mm, respectively, as clear zone in paper disc method	[25]
Antioxidant activity	Aqueous extract and 50%, 70% and 100% EtOH extract	Jangseong-gun, Jeollanam-do, Korea	DPPH radical scavenging activity of DW extract (100 μg/mL), and 50%, 70% and 100% EtOH extract (100 μg/mL) was 75.62%, and 63.91%, 40.95% and 9.38%, respectively. ABTS+ radical scavenging activity of DW extract (100 μg/mL), and 50%, 70% and 100% EtOH extract (100 μg/mL) was 57.09%, and 84.48%, 82.48% and 11.53%, respectively.	[17]
Antioxidant activity	Ethyl acetate and butanol fraction	Cheongyang-gun, Chungchungnam-do in 2019	DPPH radical scavenging activity of the ethyl acetate fraction was 559.22 μg/mL of IC_50_. ABTS radical scavenging activity of the butanol fraction was 170.18 μg/mL of IC_50_.	[26]
Dipeptidyl peptidase-4 (DPP-4) inhibitory activities			IC_50_ values of ethyl acetate fraction was 1491.65 μg/mL	
Anti-inflammatory activities	Hexane fraction	Cheongyang-gun, Chungchungnam-do in 2019	NO production inhibited 17.30% by hexane fraction (50 μg/mL) in LPS-induced RAW264.7 macrophages	[27]
Antidiabetic activity	Ethanol extract	Københans Universitet, Denmark in 2016	Drone pupae extracts showed inhibitory effects of 17.6 %, against α-amylase, and 7.2% against α-glucosidase, at a concentration of 0.5 mg/mL.	[28]
Anti-thrombotic activities			The ethanol extracts of drone pupae showed 240.7% of platelet aggregation at 0.25 mg/mL	
Anti-obesity activity	Hot air-dried and freeze-dried ones	Not mentioned	No lipase activity was detected in the control. However, the lipase activity levels of the hot air-dried and freeze-dried pupae were 2.14 ± 0.27 mU/mL and 1.06 ± 0.06 mU/mL, respectively	[29]
Anti-wrinkle effect	50% EtOH	Jangseong-gun, Jeollanam-do, Korea	Increase in collagen type I expression (46.7% and 66.7%, respectively) and decrease in MMP1 collagenase expression (36. 9% and 71.7%, respectively) were statistically significantly observed by 20 and 100 μg/mL of drone pupae extract in human dermal fibroblasts cells.	[30]
Skin whitening effect			In vitro tyrosinase activities against L-tyrosine substrate and L-DOPA substrate were inhibited 40.7% and 53.4%, respectively, by 5 mg/mL of drone pupae extract (DPE). In B16 F10 cells treated with m-melanocyte-stimulating hormone, the melanin content of 41.7% was statistically significantly decreased by 100 μg/mL of DPE	
Hair loss preventing effect	50% EtOH	Jangseong-gun, Jeollanam-do, Korea	TGF-β1 gene expression inhibited and IGF-1 gene expression recovered by 50% EtOH extract of drone pupa. In addition, TNF-α gene, IL-6 gene and caspase-3 gene were suppressed and collagen type 1 gene expression promoted by 50% EtOH extract of drone pupa.	[31]
Stimulatory effect on serum testosterone level	drone pupae powder	Wanju-gun, Jeollabuk-do, Korea	Serum testosterone level in drone pupae powder-fed SD rats increased to 2.750 ± 0.843 pg/mL compared to that (2.225 ± 0.435 pg/mL) of the control group.	[32]

## Data Availability

Data supporting reported results are available upon request.

## References

[1] Winston M.L. (1991). The Biology of the Honey Bee.

[2] Kim B.H., Yoo Y.W., Park S.J., Song W.J., Shin J.N., Oh D.H., Woo G.Y., Lee G.W., Jang Y.D., Jo G.Y. (1996). Apiology.

[3] Jeong N.G. (2011). Development and life history of honeybee. J. Korean Vet. Med. Assoc..

[4] Jeong M.K., Huh D., Lee Y.K., Lee J.M., Kim T.R. (2019). A Study on the Actual Condition of the Beekeeping Industry.

[5] Kim S.G., Woo S.O., Bang K.W., Jang H.R., Han S.M. (2018). Chemical composition of drone pupa of *Api mellifera* and its nutritional evaluation. Korean J. Apic..

[6] Kang Y., Blanco K., Davis T., Wang Y., DeGrandi-Hoffman G. (2016). Disease dynamics of honeybees with Varroa destructor as parasite and virus vector. Math. Biosci..

[7] Choi Y.S., Lee M.L., Lee M.Y., Kim H.K., Lee K.G., Yeo J.H., Woo S.O. (2009). Management for high quality drone products. Korean J. Apic..

[8] Choi Y.S. (2009). Pioneering the edibleization of honey bee drone pupae. Korea Beekeep. Bull..

[9] Ulmer M., Smetana S., Heinz V. (2020). Utilizing honeybee drone brood as a protein source for food products: Life cycle assessment of apiculture in Germany. Resour. Conserv. Recy..

[10] Evans J., Müller A., Jensen A.B., Dahle B., Flore R., Eilenberg J., Frøst M.B. (2016). A descriptive sensory analysis of honeybee drone brood from Denmark and Norway. J. Insects Food Feed.

[11] Kulma M., Tůmová V., Fialová A., Kouřimská L. (2020). Insect consumption in the Czech Republic: What the eye does not see, the heart does not grieve over. J. Insects Food Feed.

[12] Wilkinson K., Muhlhausler B., Motley C., Crump A., Bray H., Ankeny R. (2018). Australian consumers’ awareness and acceptance of insects as food. Insects.

[13] Krell R. (1996). Value-Added Products from Beekeeping.

[14] Ministry of Food and Drug Safety Status of Temporary Recognition of Standards and Specifications for Food Ingredients. 44. Honeybee Drone Pupae (*Apis mellifera* L.). https://www.foodsafetykorea.go.kr/portal/board/boardDetail.do.

[15] National Institute of Agricultural Sciences (2020). Study for New Food Raw Material Registration of Honeybee Pupa.

[16] Noh G.R., Kim D.I., Han G.Y., Cho J.H., Kwon H.W. Correlation analysis between the flowering period of black locust tree and the foraging behavior of honeybees due to climate change. Proceedings of the 32th Conference of the Apicultural Society of Korea.

[17] Kim J.E., Kim D.I., Koo H.Y., Kim H.J., Kim S.Y., Lee Y.B., Kim J.S., Kim H.H., Moon J.H., Choi Y.S. (2020). Analysis of nutritional compounds and antioxidant effect of freeze-dried powder of the honey bee (*Apis mellifera* L.) drone (pupal stage). Korean J. Appl. Entomol..

[18] Kim H.Y., Woo S.O., Kim S.G., Moon H.J., Han S.M. Analysis on the optimal production seasonal of drone pupae (*Apis mellifera* L.) for the using food materials. Proceedings of the 37th Conference and Symposium of the Apicultural Society of Korea, Online Conference.

[19] Choi H.M., Kim H.Y., Woo S.O., Kim S.G., Bang K.W., Moon H.J., Han S.M. (2019). Drying techniques and nutritional composition of drone pupae (*Apis mellifera* L.) as edible food. J. Apic..

[20] Ghosh S., Sohn H.Y., Pyo S.J., Jensen A.B., Meyer-Rochow V.B., Jung C. (2020). Nutritional composition of *Apis mellifera* drones from Korea and Denmark as a potential sustainable alternative food source: Comparison between developmental stages. Foods.

[21] Choi H.M., Han S.M., Kim H.Y., Woo S.O., Kim S.G., Bang K.W., Moon H.J. (2019). Investigation of heavy metals from honeybee drone pupa (*Apis mellifera* L) as an ingredient for novel foods. J. Apic..

[22] Kim S.G., Woo S.O., Jang H.R., Choi H.M., Moon H.J., Han S.M. (2018). Safety investigation on foodborne pathogens and mycotoxins in honeybee drone pupas. J. Food Hyg. Saf..

[23] Choi H.M., Woo S.O., Kim S.G., Bang K.W., Choi H.M., Moon H.J., Han S.M. (2019). Analysis of oxidative stability in drone pupae (*Apis mellifera* L.). J. Apic..

[24] Kim J.E., Kim S.K., Kang S.J., Kim D.Y., Koo H.Y., Kim S.Y., Kim Y.H., Choi Y.S. Toxicity evaluation of drone pupa extract on dermal cells. Proceedings of the 32th Conference of the Apicultural Society of Korea.

[25] Kim J.E., Kim S.G., Kang S.J., Kim D.I., Koo H.Y., Kim S.Y., Kim Y.H., Choi Y.S. Nutrient source analysis and functional investigation for the use of drone pupae. Proceedings of the 32th Conference of the Apicultural Society of Korea.

[26] Kim H.Y., Woo S.O., Kim S.G., Choi H.M., Moon H.J., Han S.M. (2020). Antioxidant and antihyperglycemic effects of honeybee drone pupae (*Apis mellifera* L.) extracts. J. Apic..

[27] Kim H.Y., Woo S.O., Kim S.G., Bang K.W., Choi H.M., Moon H.J., Han S.M. (2019). Anti-inflammatory activities of drone pupae (*Apis mellifera* L.) in macrophages. J. Apic..

[28] Pyo S.J., Jung C., Sohn H.Y. (2020). Platelet aggregatory and antidiabetic activities of larvae, pupae, and adult of honeybee drone (*Apis mellifera*). J. Apic..

[29] Han S.M., Woo S.O., Kim S.G., Bang K.W., Kim H.Y., Choi H.M., Moon H.J. (2019). Composition for Preventing or Treating Obesity Comprising Honeybee Drone Pupa. K.R. Patent.

[30] Kim J.E., Kim D.I., Koo H.Y., Kim H.J., Kim S.Y., Lee Y.B., Moon H.J., Choi Y.S. (2020). Pupal drone extracts for anti-wrinkle and skin-lightening materials. J. Life Sci..

[31] Kim J.E., Kim D.I., Koo H.Y., Kim H.J., Kim S.Y., Lee Y.B., Moon H.J., Choi Y.S. (2020). Evaluation of honey bee (*Apis mellifera*) drone pupa extracts on the improvement of hair loss. J. Apic..

[32] Hong I.P., Choi Y.S., Woo S.O., Han S.M., Kim H.K., Lee M.R., Nam S.H., Ha N.G. (2011). Stimulatory effect of *Cordyceps militaris* on testosterone production in male mouse. Korean J. Mycol..

[33] Kim J.E., Kim D.-I., Kim H.J., Kim S.Y., Lee Y.B., Moon H.J., Park H.G., Choi Y.S. (2020). Characteristics of hydrolysis of protein in drone pupa (*Apis mellifera* L.). J. Apic..

[34] Kang S.M., Maeng A.R., Seong P.N., Kim J.H., Cho S.H., Kim Y.S., Choi Y.S. (2018). Effect of drone pupa meal added as replacement of sodium nitrite and vitamin C on physico-chemical quality characteristics of emulsion-type sausage. Korean J. Food Nutr..

[35] Hong I.P., Lee M.Y., Kim N.S., Choi Y.S., Kim H.K., Lee M.L., Nam S.H., Lee K.G., Ha N.G. (2009). Development of entomopthogenic fungi medium by bee, drone powder. J. Apic..

[36] Xiaoming C., Ying F., Hong Z., Zhiyong C. Review of the Nutritive Value of Edible Insects. Forest Insects as Food: Humans Bite Back. Proceedings of the a Workshop on Asia-Pacific Resources and Their Potential for Development.

[37] Manditsera F.A., Lakemond C.M., Fogliano V., Zvidzai C.J., Luning P.A. (2018). Consumption patterns of edible insects in rural and urban areas of Zimbabwe: Taste, nutritional value and availability are key elements for keeping the insect eating habit. Food Secur..

[38] Belluco S., Losasso C., Maggioletti M., Alonzi C.C., Paoletti M.G., Ricci A. (2013). Edible insects in a food safety and nutritional perspective: A critical review. Compr. Rev. Food Sci. Food Saf..

[39] Ribeiro J.C., Cunha L.M., Sousa-Pinto B., Fonseca J. (2018). Allergic risks of consuming edible insects: A systematic review. Mol. Nutr. Food Res..

[40] EFSA Scientific Committee (2015). Risk profile related to production and consumption of insects as food and feed. EFSA J..

[41] Editorial Department of Euiseongdang Publishing Co. Ltd (2021). Translated ShennongBencaojing.

[42] Korean Medicine Institute (2018). Translated BenChaoGangMu.

[43] Donguibogam Committee (1999). Translated Donguibogam.

[44] Oh M.S., Kim D., Lee S. (2016). History, current status, and discussion on the future vision of *Apis cerana* beekeeping in Korea. J. Apic..

